# Hints from the Antivivisectionists

**Published:** 1914-02

**Authors:** 


### Hints from the Antivivisectionists

It often happens that the same thing is useful in different ways and that the same subject is of interest from entirely different standpoints. It occurs to us that the antivivisectionists have given the medical profession numerous hints which, properly followed up, may develop information of considerable scientific and practical humanitarian value.

1. The claim has been made repeatedly, and recently, that animal experimentation does not yield results of value to medicine, since the conditions in man and the lower animals, indeed in different species throughout the animal kingdom, are too different to warrant deductions from one animal to another. We need no present arguments to show that a large amount of our knowledge of physiology, pathology and even pharmacology does depend upon animal experimentation. But that specific and, a fortiori, generic differences in physiology and drug and disease reactions depending ultimately upon physiology exist, can not be denied. These differences are of the greatest scientific and therapeutic interest and, indeed, in so far as the standardization of drugs by animal reactions is concerned, even of economic importance. While comparative anatomy is a recognized branch of science, comparative physiology exists in a comparatively rudimentary and fragmentary state. One of the points of greatest interest in comparative physiology, is the natural immunity of certain animals against certain diseases, also the extreme variations in susceptibility, manifestation and localization of infection and the almost absolute freedom from spontaneously acquired disease in a given animal, which is, nevertheless, not immune to infection under artificially produced conditions. There does not seem to be the slightest relation of immunity and susceptibility to zoölogic relations, widely different animals showing close analogies in the case of some diseases, though not necessarily to related diseases, and conversely. Without regard to vivisectional ethics, the medical profession should realize its ignorance of comparative physiology and the great promise both of scientific grasp and practical results which a thorough investigation of this subject offers.

2. In the anxiety to refute the arguments of antivivisectionists sweeping claims have been made for the value to humanity of biologic products differing considerably in kind and for which no adequate term has yet been accepted, but all depending in some way upon reactions between disease germ and more or less susceptible animal cells. So far as antivivisection is concerned, these products are not particularly significant except for those who have an unusually high regard for the comfort and dignity of the lower animals since, generally speaking, these preparations do not involve more suffering than the use of animals to yield wool, milk, or, at most, their slaughter for meat and hides. It is unfashionable to speak of these products except with bated breath and in the highest praise, but one who will simply check them off, singly, and abide by actual accumulated experience must, we think, admit that, at present, there is only one among them that shines out with first magnitude, and this is variola vaccine, which has gained greatly in convenience and economy of use, considerably in safety but not much in actual efficiency since the introduction of modern methods. A very few others are of demonstrated value but qualified with a considerable percentage of inefficiency and the great majority are, at present, not developed to a satisfactory degree. Enough promise is given by them to warrant the hope, even the confident expectation that with a reasonable time for perfecting details and establishing standards, these remedies may prove one of the greatest parts of our armamentarium and may even supersede drugs of unquestioned value, not to mention surgical procedures. But we should not be so enthusiastic on the one point of animal experiment to create either a professional or popular confidence in unproved remedies that will prove a disappointment.

3. It occurs to us that it is quite unjustifiable to support a sweeping argument for vivisection by putting the loud pedal upon the class of substances just mentioned. It is quite conceivable that the medical profession may be driven to battle for the right to continue such experimentation, in legislatures and not merely in the way of combating some phases of popular sentiment A pretty sharp line may be drawn, from various standpoints, between the use of animals to test and ultimately to prepare these biologic disease reagents, and vivisection in the older and more literally correct sense. If a direct legislative battle ever occurs, the profession cannot hope to satisfy a critical board of inquiry as to the latter by arguments as to the results of serums and antitoxins. And, if restriction of the latter should prove to be imminent, the separation of the question into its components would prove the sincerity of the medical profession and, at the worst, would undoubtedly save the day for a really valuable and highly promising group of diagnostic and therapeutic substances.

4. We need only allude to the word pictures drawn of the torture of animals under curare. It is obvious that we can judge of the correctness of the reiterated statement as to the preferential motor action of this drug, only from records of its action on human beings. We can infer the sensation of pain by an animal only from motor reflexes; we cannot interrogate it afterward. From the analogy of other drugs it is, at least, debatable whether curare does produce paralysis of motion without affecting sensory nerves and centers. And even the identity of curare is not, we believe, definitely established. It is worth while to have definite, impartial knowledge regarding this substance whose physiologic action, at present, is stated largely in terms of antivivisection orators.

5. Without in any way suggesting or justifying serial investigation, we think the profession should consider seriously whether traumatic lesions can ever be systematized so as to furnish either a scientific or a practical relation between vulnerating causes and pathologic results.

6. In an entirely impartial and scientific spirit the inhibition of reflexes by pain should also be seriously studied. So far as any problem of physiology is concerned which has any bearing on our own field of clinical experience and observation, we are quite prepared to admit that any considerable degree of pain would absolutely vitiate any conclusion as to either motor or secretory function. In other words, an experiment without anæsthesia would introduce a degree of shock or of reflex interference such as to render it valueless. As to other branches of medicine, we do not care to venture an opinion.

7. One of the strong "talking points" of antivivisectionists is that animal experimentation is only a precursor to human experimentation. There is no denying that the latter is occasionally practiced. Only last year we cited a report of experimental implantations of gonococci. We hold that such experimentation is absolutely unjustified, especially if practiced upon patients who do not know or who do not fully realize to what they are being subjected. It is even doubtful whether experimentation with the consent of the patient is justified or whether self-experimentation, usually lauded as martyrdom to science, does not involve essentially the same ethical factors as suicide. But some qualification is necessary. Observation of secretions furnished by volunteers, as medical students, under conditions that involve no danger of life and no serious discomfort are of course to be excepted. So, too, we must except the reasonable application of well grounded hopes of benefit to patients not curable by established means, as well as the introduction into therapeutics of new remedies whose pharmacologic action and safety have been established by purely experimental means.

